# Decoding Alzheimer’s in the age of genome-wide analyses

**DOI:** 10.1186/1750-1326-8-S1-O1

**Published:** 2013-09-13

**Authors:** Rudolph E Tanzi

**Affiliations:** 1 Massachusetts General Hospital/Harvard Medical School, Charlestown, MA, USA

## 

Alzheimer’s disease (AD) is strongly influenced by genetic factors as evidenced by numerous family and twin studies. Over the past two decades, others and we have co-discovered three early-onset familial AD genes, *APP*, *PSEN1*, and *PSEN2*, which can carry any of >200 fully penetrant mutations characterized by mendelian inheritance. For late-onset AD, the most prevalent and highest impact risk factor is the epsilon 4 variant of *APOE*, which increases risk by 3.7-fold in the heterozygous state and >10-fold when two copies are inherited. As part of our Alzheimer’s Genome Project funded by the Cure Alzheimer’s Fund and the NIMH, we have been identifying novel AD candidate genes. In Bertram *et al.* (2008), we reported genome-wide association of AD with several loci including the spinocerebellar ataxia 1 gene, *ATXN1*, and the SIGLEC3 gene, *CD33.* Functional studies of *the ATXN1* gene carried out both *in vitro* and *in vivo* in *ATXN1* knockout mice show that *ATXN1* can regulate Aβ levels via modulation of β-secretase. *CD33* is just one of several genes (*TREM2*, *CR1*, etc) involved in the innate immune system of the brain that have been associated with AD risk. In addition, we discovered association of AD with *ADAM10*, which encodes the major α-secretase in the brain. Re-sequencing of this gene in the best associated AD families led to two rare mutations that tightly co-segregated with AD in seven (of 1000) AD families screened, all with average age of onset ~70 years. We have shown both mutations to impair *ADAM10* non-amyloidogenic cleavage of APP *in vitro* and in transgenic mice, including double trangenics with AD (tg2576) mice. Thus, these are the first rare, highly penetrant mutations reported for late-onset AD. We have also carried out genome-wide association studies on >800 well-characterized late-onset AD families (NIMH and NIA-LOAD samples) using Affymetrix genotyping arrays containing either one million (6.0) or 20,000 coding SNPs. More recently, we have performed whole genome sequencing (Illumina, Hiseq) on over 1500 samples from AD families/sibships. The elucidation of the genes and functional variants influencing risk for AD should continue to enhance our understanding of AD etiology and pathogenesis. Ultimately, these genes will be used to predict risk for AD and guide novel the development of therapies for *the* effective treatment and prevention of this terrible disease.

